# Autism Knowledge Assessments: A Closer Examination of Validity by Autism Experts

**DOI:** 10.1007/s10803-024-06293-7

**Published:** 2024-04-07

**Authors:** Camilla M. McMahon, Maryellen Brunson McClain, Savannah Wells, Sophia Thompson, Jeffrey D. Shahidullah

**Affiliations:** 1 Department of Social and Behavioral Sciences, Miami University, 1601 University Blvd., Hamilton, OH 45011 USA; 2 Department of Psychology, Miami University, 90 North Patterson Avenue, Oxford, OH 45056 USA; 3 Department of Counseling and Educational Psychology, Indiana University Bloomington, 201 N. Rose Avenue, Bloomington, IN 47405 USA; 4 Department of Psychiatry and Behavioral Sciences, Dell Medical School, University of Texas at Austin, 1601 Trinity Blvd, Austin, TX 76018 USA

**Keywords:** Autism knowledge, Substantive validity, Accuracy, Ambiguity, Heterogeneity of the autism spectrum, Autism experts

## Abstract

Purpose: The goal of the current study was to conduct a substantive validity review of four autism knowledge assessments with prior psychometric support (Gillespie-Lynch in J Autism and Dev Disord 45(8):2553–2566, 2015; Harrison in J Autism and Dev Disord 47(10):3281–3295, 2017; McClain in J Autism and Dev Disord 50(3):998–1006, 2020; McMahon in Res Autism Spectr Disord 71:101499, 2020). 69 autism experts who served on the editorial board of one or more peer-reviewed autism journals evaluated the accuracy and ambiguity of autism knowledge questions. 34% of the questions were flagged as “potentially problematic” for accuracy, and 17% of the questions were flagged as “potentially problematic” for ambiguity. Autism expert feedback revealed three themes across ambiguous questions: (1) an oversimplification of mixed or still-evolving research literature, (2) an insufficient recognition of the heterogeneity of the autism spectrum, and (3) a lack of clarity in the question/answer prompt. Substantive validity of future autism knowledge assessments should be carefully evaluated via feedback from a diverse group of autism experts and/or potential respondents. Potentially problematic questions can be removed or modified to improve the validity of autism knowledge assessments.

There has been a growing interest in measuring autism knowledge in recent years, such that a variety of different autism knowledge assessments have been developed. In their literature review, Harrison et al. (2017b) identify 44 different autism knowledge assessments. When rating the psychometric properties of these 44 assessments, only 5 assessments showed evidence of strong psychometric support, defined by the authors as careful evaluation of at least one aspect of both reliability and validity. As such, the majority (87%) of autism knowledge assessments did not show evidence of strong psychometric support, and even those knowledge assessments classified as having strong psychometric support did not necessarily show robust evidence of reliability and validity across multiple indices. Autism knowledge is an important construct to measure, as it allows researchers to assess awareness of autism in the general population, the development of autism expertise in professional populations, and the reduction of autism-related stigma and misconceptions. While additional autism knowledge assessments have been developed since this literature review (Harrison et al., 2017b), this review highlights the need for more rigorous evaluation of the psychometric properties of autism knowledge assessments.

## Assessing the Validity of Autism Knowledge Assessments

Flake et al. (2017) outline three phases of construct validity that should be evaluated when developing assessments: substantive validity (e.g., expert review, cognitive interviewing), structural validity (e.g., reliability, confirmatory factor analysis), and external validity (e.g., correlations with other scales, ability to predict dependent variables). Gehlbach and Brinkworth (2011) note that the initial phase of substantive validity is often overlooked or cursorily addressed in scale development, in favor of conducting statistical analyses that assess structural validity. However, the substantive validity phase allows for valuable feedback from both experts and potential respondents as to the accuracy and clarity of assessment questions. When conducting an expert review, for instance, established experts in the research literature examine assessment items on pertinent criteria, such as relevance and clarity, and have an opportunity to write-in additional comments about the assessment items. This allows researchers to identify problems with an assessment item, from insufficient support in the research literature to ambiguity in the question prompt, that may not be discovered in later statistical analyses.

When soliciting feedback from multiple respondents, Herzog and Hertwig (2011) recommend collecting information from “a diverse set of respondents that have been exposed to different information environments”. In other words, expert feedback will be most helpful when experts are least similar to one another (Afflerbach et al., 2021). When asking autism experts to evaluate autism knowledge questions, a heterogeneous pool of participants should be solicited. If possible, autism experts should vary in terms of personal/professional expertise (e.g., autistic individuals^1^, parents, clinicians), disciplinary expertise (e.g., neuroscience, special education, psychology), and racial/cultural/international expertise (e.g., Black, British, Latin American). Ideally, given the divergent theoretical perspectives in autism research and advocacy today (e.g., opposing position statements from the Autistic Self Advocacy Network (2024) and the National Council on Severe Autism (2024)), autism experts should also represent different theoretical and advocacy perspectives to maximize intellectual diversity. If a content review is conducted by a relatively homogenous group of experts, potential difficulties related to assessment accuracy, clarity, and terminology may be missed.

## Current Study

Given the need to conduct a more rigorous analysis of the psychometric properties of autism knowledge assessments, the current study investigates the validity of four autism knowledge assessments currently published in the research literature (Gillespie-Lynch et al., 2015; Harrison et al., 2017a; McClain et al., 2020; McMahon et al., 2020). This study aims to conduct a substantive validity review of these assessments, relying on the expertise of established autism researchers and clinicians across a diverse array of disciplinary backgrounds. The four autism knowledge assessments examined in this study were chosen as (1) they already have modest or strong published evidence of psychometric support, as operationalized by Harrison et al. (2017b), (2) they are authored by different individuals at different universities, representing a greater diversity of autism expertise, (3) they are fairly representative of autism knowledge assessments in the broader autism knowledge literature, and (4) they have minimal overlapping content and format, with different assessments emphasizing different content areas (e.g., basic vs. advanced autism knowledge) or using different response options (e.g., Likert scale, agree/disagree, multiple choice questions). As the four assessments examined in this study vary in terms of content difficulty and response format, the current study does not directly compare psychometric properties across the assessments. Rather, this study aims to solicit expert feedback on a representative sample of autism knowledge questions in the research literature, with the goal of improving measurement of autism knowledge across both current and future assessments.

## Methods

### Participants

Individuals on the editorial board for one or more of the following eight autism-focused journals were invited to participate in the current study: (1) *Autism*, (2) *Autism and Developmental Language Impairments*, (3) *Autism in Adulthood*, (4) *Autism Research*, (5) *Focus on Autism and Other Developmental Disabilities*, (6) *Journal of Autism and Developmental Disorders*, (7) *Molecular Autism*, and (8) *Research in Autism Spectrum Disorders*. A list of editorial board members (e.g., editors, associate editors, editorial board members) was compiled from the journal websites in fall 2021. Individuals were excluded from participation if they co-authored one of the autism knowledge assessments examined in the current study (i.e., Gillespie-Lynch et al., 2015, 2019; Harrison et al., 2017a; McClain et al., 2020; McMahon et al., 2020).

In total, 467 potential participants were deemed eligible for the study. Potential participants were informed of the study through a recruitment e-mail or message that explained the purpose of the study and instructions for participating. E-mails of eligible participants were located via Google search and previous publications. If e-mails were inaccessible, then eligible participants were messaged through ResearchGate (a research sharing platform). Contact information for three potential participants could not be identified. In addition, obituary notices were located for two potential participants. After an initial contact message, potential participants were sent up to two reminder messages regarding study participation. After sending initial and reminder recruitment messages, 72 participants consented to participate in the research study and began data collection. Participants who only contributed demographic information and did not respond to any of the autism knowledge questions (*n* = 3) were excluded from data analyses. The final sample consisted of 69 participants who completed or partially completed the research study, resulting in a 14.8% response rate (see Table 1 for participant characteristics). No participation incentives were offered.

**Table 1:** Participant characteristics

Variable	*n*	%	*M*	*SD*
Role Emphasis^a^				
Clinician	43	62		
Researcher	68	99		
Disciplinary Area^a^				
Education (e.g., special education, general education)	20	29		
Behavioral Health (e.g., psychology, applied behavioral analysis, social work)	34	49		
Medicine (e.g., nursing, pediatrics, psychiatry)	12	17		
Allied Health (e.g., speech-language pathology, occupational/physical therapy, audiology)	7	10		
Biological Sciences (e.g., genetics, epidemiology, neuroscience, immunology)	17	25		
Age (years)			53.46	11.65
Years of Experience in Autism			26.71	10.42
Personal Expertise in Autism			1.45	1.32
Experience with DSM-5 Severity Levels				
Severity Level 1 (Individuals who require support)			4.25	1.24
Severity Level 2 (Individuals who require substantial support)			4.12	1.21
Severity Level 3 (Individuals who require very substantial support)			3.68	1.35

Gender, ethnicity, and race data are not presented in this table in order to protect participant confidentiality, given that the participant pool contains a relatively small group of individuals from publicly searchable editorial boards. DSM-5 = Diagnostic and Statistical Manual of Mental Disorders, Fifth Edition (American Psychiatric Association, 2013, 2022).

^a^Participants were allowed to select more than one category, such that the categories do not total 100%.

### Measures

#### Demographics Questionnaire

 Participants completed a brief demographics questionnaire collecting information on gender, race, ethnicity, age, years of experience in the field of autism, identification as a researcher and/or clinician, and general area(s) of disciplinary expertise. In addition, participants reported on their personal expertise in autism, with their personal expertise score as the total number of statements checked out of 5 personal expertise statements (e.g., “I am autistic”; “an immediate family member is autistic”). Finally, participants used a Likert scale (1 = no experience to 5 = substantial experience) to indicate their expertise with autistic individuals at Severity Level 1 (“requiring support”), Severity Level 2 (“requiring substantial support”), and Severity Level 3 (“requiring very substantial support”), as operationalized in the Diagnostic and Statistical Manual of Mental Disorders, Fifth Edition (DSM-5; American Psychiatric Association, 2013, 2022).

#### Gillespie-Lynch et al. (2015); Autism Awareness Scale

 This assessment includes 13 questions with a 5-point Likert scale response format from *strongly disagree* to *strongly agree*. The assessment is a contemporary version of the autism knowledge assessment originally developed by Stone (1987), and in the current study, the last assessment question on empathy is updated to reflect the revision made by Gillespie-Lynch et al. (2019). The assessment has marginally acceptable reliability for participants in the U.S. (α = 0.68; Gillespie-Lynch et al., 2019). In the current study, participants received 1 full point for responding with the correct answer (*strongly agree* or *strongly disagree*) and received 0.75, 0.5, 0.25, and 0 points respectively for the other response choices on the Likert scale.

#### Harrison et al. (2017a); Autism Stigma and Knowledge Questionnaire

The final version of this assessment contains 48 autism knowledge statements with a response format of *agree*/*disagree/don’t know*. Autism knowledge experts from a variety of countries reviewed the pilot version of this questionnaire for face, construct, and cross-cultural validity, and the questions with the highest validity scores and clearest answers were retained in the final questionnaire. This questionnaire shows good reliability (*α* = 0.88).

#### McClain et al. (2020); Autism Spectrum Knowledge Scale, Professional Version—Revised

 This assessment contains 25 multiple choice questions with 4 content answer choices. As the assessment is designed to measure autism knowledge in professional populations, not a lay audience, it includes more challenging content questions, including items assessing knowledge of prevalence, evidence-based interventions, and diagnosis. Three autism experts reviewed the assessment and provided feedback to improve face validity during development, and the assessment shows acceptable reliability (KR20 = .70).

#### McMahon et al. (2020); Autism Symptomatology Knowledge Assessment

 This assessment features 25 symptoms of conditions operationalized in the DSM-5, including 10 symptoms of autism and 15 symptoms of other clinical disorders, such as depression and ADHD. For each symptom, participants are asked to indicate whether the symptom can be used to diagnose an individual with autism spectrum disorder (*yes* or *no*). To ensure content validity, all symptoms were taken from the DSM-5 (American Psychiatric Association, 2013, 2022). The overall assessment shows acceptable reliability (*α* = 0.77).

### Procedure

This study was declared exempt by the university Institutional Review Board. Participants completed the study online using Qualtrics survey software. After completing the demographics survey, participants viewed the autism knowledge questions. The 111 autism knowledge questions, across the four assessments, were divided into two distinct sets of questions. Each set contained approximately half of the questions from each autism knowledge assessment, totaling 55 or 56 questions. Participants were randomly assigned one set of questions to complete. For each autism knowledge question, participants were asked to indicate which answer they judged to be the most valid. As participants were autism experts across a variety of disciplines (from medicine to education), participants also had the option of not answering an autism knowledge question if they believed the question was outside of their area of expertise. Next, participants were provided with the “correct” answer to the question, as indicated by the assessment authors, and asked to rate the ambiguity of the question/answer on a scale from 1 (*not at all ambiguous*) to 5 (*extremely ambiguous*). If the ambiguity was rated 3 (*moderately ambiguous*) or higher, participants were asked to provide an explanation for why the question/answer was ambiguous.^2^ The following description of the term “ambiguity” was provided to participants in the study directions:

*There are many different reasons why a question and its corresponding answer could be judged to be ambiguous. For example:*

- *The terms in a question may not be clearly defined.*
- *The terms could be interpreted differently by different stakeholders in the autism community.*
- *The terms may differ in meaning across various professional disciplines.*
- *The terms may not apply equally to all individuals across the autism spectrum.*
- *There may not be a clear correct answer to the question.*
- *More than one answer to the question could be reasonably argued to be accurate.*

### Data Analysis Plan

Autism knowledge questions were flagged as “potentially problematic” for accuracy if less than 90% of autism experts judged that the “correct” answer provided by the assessment authors was actually correct and/or the average ambiguity rating for the question exceeded 2 (*slightly ambiguous*). Participants’ qualitative comments regarding question/answer ambiguity were reviewed, and themes surrounding these qualitative comments were identified.

## Results

### Accuracy and Ambiguity

Thirty-four percent of the questions (*n* = 38) were flagged as “potentially problematic” for accuracy (see Table 2). Of these questions, six questions had mean accuracy scores lower than 50%, indicating that the majority of autism experts disagreed with the answer indicated to be “correct” by the assessment authors. Seventeen percent of the questions (*n* = 19) were flagged as “potentially problematic” for ambiguity (see Table 2). There was considerable disagreement among autism experts about the ambiguity of the questions. All 111 autism knowledge questions were given an ambiguity rating of 1 (*not at all ambiguous*) by at least one autism expert. At the same time, 53% of the questions (*n* = 59) were given an ambiguity rating of 5 (*extremely ambiguous*) by at least one autism expert. Fourteen percent of the questions (*n* = 15) were flagged as “potentially problematic” for both accuracy and ambiguity.

**Table 2:** Autism knowledge assessment questions flagged as potentially problematic for accuracy and/or ambiguity

Reference (Question)	Question	Accuracy			Ambiguity		
		*n*	*M*	*SD*	*n*	*M*	*SD*
Gillespie-Lynch et al., 2015 (#3)	People with autism are deliberately uncooperative	32	0.86	0.25	–	–	–
	• **Strongly Disagree**						
	• Disagree						
	• Neither Agree nor Disagree						
	• Agree						
	• Strongly Agree						
Gillespie-Lynch et al., 2015 (#4)	Children with autism can grow up to go to college and marry	31	0.83	0.23	–	–	–
	• Strongly Disagree						
	• Disagree						
	• Neither Agree nor Disagree						
	• Agree						
	• **Strongly Agree**						
*Gillespie-Lynch et al., 2015 (#6)	Autism can be diagnosed as early as 15 months of age	29	0.65	0.28	32	2.09	1.17
	• Strongly Disagree						
	• Disagree						
	• Neither Agree nor Disagree						
	• Agree						
	• **Strongly Agree**						
Gillespie-Lynch et al., 2015 (#7)	With the proper treatment, most children diagnosed with autism eventually outgrow the disorder	31	0.90	0.20	–	–	–
	• **Strongly Disagree**						
	• Disagree						
	• Neither Agree nor Disagree						
	• Agree						
	• Strongly Agree						
*Gillespie-Lynch et al., 2015 (#8)	People with autism show affection	31	0.80	0.27	31	2.10	1.27
	• Strongly Disagree						
	• Disagree						
	• Neither Agree nor Disagree						
	• Agree						
	• **Strongly Agree**						
Gillespie-Lynch et al., 2015 (#9)	Most people with autism have low intelligence	31	0.89	0.24	–	–	–
	• **Strongly Disagree**						
	• Disagree						
	• Neither Agree nor Disagree						
	• Agree						
	• Strongly Agree						
Gillespie-Lynch et al., 2015 (#10)	Children with autism grow up to be adults with autism.	32	0.88	0.14	–	–	–
	• Strongly Disagree						
	• Disagree						
	• Neither Agree nor Disagree						
	• Agree						
	• **Strongly Agree**						
*Gillespie-Lynch et al., 2015 (#11)	People with autism tend to be violent	32	0.86	0.23	32	2.06	1.32
	• **Strongly Disagree**						
	• Disagree						
	• Neither Agree nor Disagree						
	• Agree						
	• Strongly Agree						
Gillespie-Lynch et al., 2015 (#12)	People with autism are generally disinterested in making friends	31	0.82	0.21	–	–	–
	• **Strongly Disagree**						
	• Disagree						
	• Neither Agree nor Disagree						
	• Agree						
	• Strongly Agree						
*Gillespie-Lynch et al., 2019 (#13)	People with autism care about and feel the pain of those who are suffering	30	0.78	0.21	33	2.36	1.19
	• Strongly Disagree						
	• Disagree						
	• Neither Agree nor Disagree						
	• Agree						
	• **Strongly Agree**						
*Harrison et al., 2017a (#9)	It is important that all children diagnosed with autism receive some form of special education services at school	33	0.58	0.50	33	2.67	1.56
	• Disagree						
	• **Agree**						
*Harrison et al., 2017a (#20)	Children with autism need extra help to learn	30	0.87	0.35	31	2.35	1.28
	• Disagree						
	• **Agree**						
Harrison et al., 2017a (#22)	The earlier treatment of autism starts, the more effective it tends to be	31	0.87	0.34	–	–	–
	• Disagree						
	• **Agree**						
*Harrison et al., 2017a (#28)	Most children with autism may not look at things when you point at them	30	0.80	0.41	31	2.32	1.11
	• Disagree						
	• **Agree**						
Harrison et al., 2017a (#34)	Many children with autism repeatedly spin objects or flap their arms	30	0.83	0.38	–	–	–
	• Disagree						
	• **Agree**						
*Harrison et al., 2017a (#35)	Autism is a communication disorder	31	0.32	0.48	31	3.06	1.29
	• **Disagree**						
	• Agree						
Harrison et al., 2017a (#36)	Autism occurs more commonly among higher socioeconomic and educational levels	30	0.83	0.38	–	–	–
	• **Disagree**						
	• Agree						
*Harrison et al., 2017a (#38)	Behavior therapy is an intervention most likely to be effective for children with autism	31	0.84	0.37	32	2.34	1.36
	• Disagree						
	• **Agree**						
*Harrison et al., 2017a (#41)	A lot of children with autism have problems with being aggressive or hyperactive	32	0.53	0.51	32	2.50	1.27
	• Disagree						
	• **Agree**						
Harrison et al., 2017a (#44)	Many children with autism have difficulty using everyday language to communicate their needs	32	0.88	0.34	–	–	–
	• Disagree						
	• **Agree**						
Harrison et al., 2017a (#51)	Autism is caused by God or a supreme being	–	–	–	31	2.03	1.47
	• **Disagree**						
	• Agree						
McClain et al., 2020 (#1)	Current estimates suggest that autism spectrum disorder affects approximately _____ percent of the population?	29	0.90	0.31	–	–	–
	• Less than 1%						
	• **Between 1-2%**						
	• Between 3%-5%						
	• Greater than 5%						
McClain et al., 2020 (#2)	Autism spectrum disorder is approximately _____ times more likely to be diagnosed in boys than girls	30	0.83	0.38	–	–	–
	• 0 times (it is diagnosed in boys and girls equally)						
	• 2 times						
	• **4 times**						
	• 5 times						
McClain et al., 2020 (#3)	Please select the risk factor that is not associated with autism spectrum disorder	31	0.87	0.34	–	–	–
	• Advanced paternal age						
	• Family history of autism spectrum disorder						
	• **Vaccines**						
	• Pollution						
McClain et al., 2020 (#5)	Approximately__percent of individuals with autism spectrum disorder also have an intellectual disability	29	0.45	0.51	–	–	–
	• 25%						
	• **33%**						
	• 50%						
	• 66%						
McClain et al., 2020 (#8)	In the United States public school setting, children with autism spectrum disorder may be eligible for special education if	25	0.88	0.33	–	–	–
	• A medical doctor (e.g., pediatrician) independently determines it is necessary						
	• **The students’ disability has an educational impact**						
	• The student says they want special education services						
	• Their teacher independently decides it is necessary						
McClain et al., 2020 (#10)	Which of the following is not an evidence-based intervention for individuals with autism spectrum disorder?	28	0.86	0.36	–	–	–
	• Video modeling						
	• Applied behavior analysis						
	• Pivotal response training						
	• **Equine therapy**						
McClain et al., 2020 (#12)	Up to __ percent of individuals with autism spectrum disorder also have a co-occurring mental health diagnosis	30	0.50	0.51	–	–	–
	• 20%						
	• 30%						
	• 50%						
	• **70%**						
McClain et al., 2020 (#13)	When assessing a bilingual child for autism spectrum disorder, it is important to obtain language information regarding:	29	0.83	0.38	–	–	–
	• The child’s native language development						
	• The child’s English language development						
	• **The child’s English language development and native language development**						
	• Information regarding the English language development of the child only between birth to 2 years of age						
*McClain et al., 2020 (#17)	Which of the following is not an example of a restricted and repetitive pattern of behavior or interest	31	0.48	0.51	33	2.27	1.35
	• Stereotypic motor movements						
	• Strict adherence to a routine						
	• Echolalia						
	• **Obsessive behaviors**						
McClain et al., 2020 (#20)	With regard to reading, children with autism spectrum disorder are more likely to experience difficulties with:	25	0.88	0.33	–	–	–
	• Reading speed						
	• **Reading comprehension**						
	• Phonological awareness						
	• Reading accuracy						
*McClain et al., 2020 (#21)	In general in the United States, diagnostic tools for autism spectrum disorder are:	21	0.43	0.51	32	2.09	1.20
	• Standardized in many other languages besides English						
	• Translated into many other languages, but not standardized in this format						
	• Include bilingual learners in the standardization sample						
	• **Sometimes translated into Spanish, but not standardized in this format**						
McClain et al., 2020 (#22)	By the age of _____ years a diagnosis of autism spectrum disorder can be reliably diagnosed	32	0.69	0.47	–	–	–
	• **2**						
	• 4						
	• 6						
	• 8						
*McClain et al., 2020 (#23)	The majority of children with autism spectrum disorder are diagnosed after age:	30	0.30	0.47	32	2.53	1.41
	• 3 years						
	• **4 years**						
	• 5 years						
	• 6 years						
McClain et al., 2020 (#24)	Autism prevalence rates across the globe:	28	0.71	0.46	–	–	–
	• Are highly consistent with the prevalence rates in the United States						
	• **Are extremely varied**						
	• Are much higher in African countries than all other countries						
	• Are higher in countries where English is the national language						
*McClain et al., 2020 (#25)	Clinicians must identify all but _____ when making a diagnosis of autism spectrum disorder?	25	0.40	0.50	32	3.16	1.55
	• If disorder occurs with or without intellectual impairment						
	• If the disorder is associated with a known medical or genetic condition or environmental factor						
	• **If the disorder is associated with known sensory sensitivities**						
	• If the disorder occurs with or without accompanying language impairment						
McMahon et al., 2020 (#3)	Can this symptom be used to diagnose an individual with Autism Spectrum Disorder? - Difficulties adjusting behavior to suit various social contexts	31	0.87	0.34	–	–	–
	• **Yes**						
	• No						
*McMahon et al., 2020 (#4)	Can this symptom be used to diagnose an individual with Autism Spectrum Disorder? - Poorly integrated verbal and nonverbal communication	30	0.83	0.38	33	2.18	1.33
	• **Yes**						
	• No						
McMahon et al., 2020 (#9)	Can this symptom be used to diagnose an individual with Autism Spectrum Disorder? - Insistence on sameness	29	0.90	0.31	–	–	–
	• **Yes**						
	• No						
McMahon et al., 2020 (#15)	Can this symptom be used to diagnose an individual with Autism Spectrum Disorder? - Failure of normal back-and-forth conversation	–	–	–	31	2.19	1.11
	• **Yes**						
	• No						
McMahon et al., 2020 (#19)	Can this symptom be used to diagnose an individual with Autism Spectrum Disorder? - Abnormalities in eye contact and body language	–	–	–	31	2.23	1.12
	• **Yes**						
	• No						
McMahon et al., 2020 (#22)	Can this symptom be used to diagnose an individual with Autism Spectrum Disorder? - Inflexible adherence to routines	–	–	–	32	2.03	1.03
	• **Yes**						
	• No						

“Correct” answers, as provided by the assessment authors, are bolded. Ambiguity and accuracy statistics are only provided when the question meets the requirements for a “potentially problematic” question. Accuracy is rounded to two decimal points. Assessment questions with an accuracy of 0.90 on this table had a mean accuracy greater than 0.895 but less than 0.90; therefore, these questions were still flagged as “potentially problematic”.

*Question was flagged as “potentially problematic” for both accuracy and ambiguity.

### Qualitative Themes

After reviewing participants’ qualitative comments regarding the ambiguity of the autism knowledge questions, the following three themes were identified (see Table 3 for example participant responses):

**Table 3:** Example qualitative responses describing why the autism knowledge question and/or answer is ambiguous

Reference (Question)	Question	Example responses: Why is the Question and/or Answer Ambiguous?
**Theme #1: Disagreement/Lack of Clarity in the Research Literature**		
Harrison et al., 2017a (#22)	The earlier treatment of autism starts, the more effective it tends to be. • Disagree • **Agree**	“What is early? There is not much evidence that intervention before two is effective or feasible. Some would also argue there is not yet definitive evidence for the efficacy of preschool interventions in general”
		“This is ambiguous as some treatments will help certain individuals but not others, and some approaches may be actively deleterious, or positive for some aspects of functioning while impairing others”
		“It depends on what they mean by treatment, on their definition of autism, and who they are targeting. Many autistic individuals are diagnosed only later in life. What is the treatment that we are talking about for these folks? The support needed may very be available only in later life and may be quite effective”
		“I knew the answer would be considered true, but I don't think it is! There are huge questions about what 'effective' intervention is, whether autism needs 'treatment' etc”
Harrison et al., 2017a (#35)	Autism is a communication disorder. • **Disagree** • Agree	“This is quite ambiguous, since communication challenges are part of ASD. It's not clear, then, if they mean a formally-defined Communication Disorder (e.g., in DSM or ICD; it's not), or a disorder that impacts communication (it is)”
		“the use of the word communication is clearly ambiguous. The core feature of ASD is that it is a social communication disorder. It is NOT a language disorder. I think the instrument authors need to consider the difference between language and communication. Failure of appropriate verbal and nonverbal social communication is at the core of all diagnoses of ASD!”
		“It is considered to be a social communication disorder and is described as such on the home page of ASHA https://www.asha.org/public/speech/disorders/autism/”
		“How did you determine that disagree was the answer, given the fact that all people diagnosed with an ASD must have some level of social communication deficits as defined in the DSM-5? ASD very definitely is a communication disorder”
McClain et al., 2020 (#2)	Autism spectrum disorder is approximately _____ times more likely to be diagnosed in boys than girls. • 0 times (it is diagnosed in boys and girls equally) • 2 times • **4 times** • 5 times	“Since many scientists reference the number 4–5 times more likely in boys than girls, this may result in confusion between those two numbers”
		“This question assumes that the person is aware of only the pediatric literature and not the literature supporting diagnosis among females in adulthood and missed diagnosis in adulthood resulting in self-identification”
		“Actually.. this is 3:1. loomes et al 2019. And ambiguous because you don’t mention within which level of iq or countries. Varies a bit across studies and samples. And age”
		“4 times seems to be the most commonly stated figure, but I'm not sure how accurate it is currently with the increased focus on diagnosis in girls and women”
McClain et al., 2020 (#12)	Up to __ percent of individuals with autism spectrum disorder also have a co-occurring mental health diagnosis • 20% • 30% • 50% • **70%**	“There are different estimates for kids, teens, and adults; also different estimates based on verbal/cognitive abilities”
		“The rates of specific mental health problems / symptoms vary greatly in people with ASD and I'm presuming that 70% is in regard to anxiety. Therefore, we should not be including all forms of mental health problems and their different causes, trajectories and treatability under a single rubric of ' mental health' ”
		“Some studies show higher rates than 70% if you look at life-time comorbidity.”
		“I don't think the literature is really clear on the prevalence of co-occurring conditions. Some studies were done in psychiatric clinics and thus would have an ascertainment bias and result in artificially higher prevalence rates. To answer this question, only epidemiological/community based studies should be used and when they are, I don't think the rates are 70%”
**Theme #2: Not Fully Acknowledging the Heterogeneity of the Autism Spectrum**		
Gillespie-Lynch et al., 2015 (#11)	People with autism tend to be violent. • **Strongly Disagree** • Disagree • Neither Agree nor Disagree • Agree • Strongly Agree	“Some people with autism can be violent - and self-injury can reasonably be considered a form of "violence" that is important to identify and address in a subset of the population. So, while it is certainly not correct to say that people with autism (as a general matter) reliably tend to be violent, it remains important to realize that some people can be - and we need to identify better ways to help these individuals”
		“Questions that require generalizations to be made are problematic”
		“In practice areas and with parents, aggression, disruption, or SIB is often termed "violent". It may be better to describe this in other terms related to "intentional harm of others", "committing assault" or something similar”
		“I can't say "Strongly disagree" because the truth is that a lot of people with autism who face barriers to communicating their needs and feelings do manifest violent outbursts. Violence is not a *general* feature of autism, because those people with autism who can communicate don't generally have such outbursts. But my parents have suffered the bite injuries that show that my autistic brother could indeed be very violent. There is no one answer to this question that covers people with autism in general. If 'People with autism' were qualified with 'Some' then the answer would be Strongly Agree. If the qualification were 'All' then the answer would be Strongly Disagree”
Gillespie-Lynch et al., 2015 (#12)	People with autism are generally disinterested in making friends. • **Strongly Disagree** • Disagree • Neither Agree nor Disagree • Agree • Strongly Agree	“The distinction between strongly disagree and disagree is nuanced. I went with strongly to avoid stigmatizing/overgeneralizing about autism, but there are some autistic people who are not interested in making friends just as some non-autistic people are not interested in making friends”
		“Methods of assessing whether or not someone is disinterested in making friends as opposed to lacking the capacity to engage with others are not well established”
		“It is difficult to answer a question that is based on a generalization. Not all autistic people are the same”
		“again, not all people with autism are socially motivated. The question does not allow for heterogeneity to be the true response”
Harrison et al., 2017a (#20)	Children with autism need extra help to learn. • Disagree • **Agree**	“Yes, they often need all sorts of supports and accommodations and I fight really hard to make those available, but what do you mean by "help" and in what circumstances? If you use a social model of disability, an autistic child growing up in an autistic family and going to a school that was run by autistic people, with autistic teachers and autistic students may do just fine and an NT kid would need "extra help"”
		“To learn what? Some are extremely academically able but have difficulties learning other things such as social skills”
		“The question does not provide for nuance. SOME individuals with autism will need extra to learn in SOME areas, but not all individuals need help and not in all areas”
		“Again, overgeneralization. I know brilliant autistic people who need 1:1 support to communicate and access education and those whose education was entirely self-taught alone in a library with no help at all”
McClain et al., 2020 (#22)	By the age of _____ years a diagnosis of autism spectrum disorder can be reliably diagnosed. • **2** • 4 • 6 • 8	“What does 'reliably' mean? Many people question if it's only reliable for subsets of autistic people (e.g., not those who come from different minority ethnic backgrounds). There is a bit more complexity behind this than the question implies”
		“Not all children can be diagnosed by age 2. Some can be, but many may not manifest sufficient symptoms to meet full criteria for disorders status”
		“Although I know 2 years old is accepted as the age when a "reliable diagnosis" can be made, this wording suggests that every autistic person can be "reliably diagnosed" at 2, which is definitely not the case”
		“Again, no allowing for heterogeneity. Diagnosis can not be made by 2 in all kids with ASD”
**Theme #3: Lack of Clarity in the Question/Answer Prompt**		
Harrison et al., 2017a (#41)	A lot of children with autism have problems with being aggressive or hyperactive. • Disagree • **Agree**	“What does 'a lot of' mean? Most? the majority?”
		“what behaviours classify as aggressive and hyperactive may be unclear”
		“The term "a lot" is very ambiguous. How much is "A lot." Also aggression and hyperactivity are two different conditions. This is a double barreled question”
		““A lot” is a very ambiguous frequency. I interpret it as meaning "most/the majority of", and I'm not sure there's evidence that most autistic children are aggressive, although I would agree that many/the majority have co-occurring ADHD. To "have problems with" aggression or hyperactivity is different than simply being aggressive or hyperactive. A child who is hyperactive might be perfectly happy with their hyperactivity, but the people around them might have problems!”
McClain et al., 2020 (#25)	Clinicians must identify all but _____ when making a diagnosis of autism spectrum disorder? • If disorder occurs with or without intellectual impairment • If the disorder is associated with a known medical or genetic condition or environmental factor • **If the disorder is associated with known sensory sensitivities** • If the disorder occurs with or without accompanying language impairment	“The question is worded strangely (all of the following EXCEPT might be clearer), and the definition of "clinicians" is broad. For a clinician using the DSM and/or ADOS/ADI-R, intellectual impairment, sensory symptoms, and language impairment are all in the DSM5 criteria. While I see that known genetic cases should be considered, it doesn't actually impact the diagnostic procedures which are based on behavior”
		“The authors of this question are looking only at the words "Specify if" at the end of the DSM-5 definition, and not at the final (#4) criterion within section B of that definition. Defining the individual's symptoms entails specifying those symptoms! So actually all these aspects must be specified; there is no right answer amongst these multiple choices”
		“Sensory sensitivities are part of the symptom profile of the Restricted and Repetitive Behavior dimension of ASD. So in any diagnostic work-up a clinician would evaluate whether they are part of the presenting picture. Only qualified clinicians (MDs) would be competent in ruling in or out medical or genetic conditions. So "Clinicians must identify..." is to broad to include that as a "must" for making a DX of ASD”
		“I do not understand the question - the construct 'clinicians must identify all but if the disorder occurs with or without intellectual impairment' makes no grammatical sense to me... etc”
McMahon et al., 2020 (#9)	Can this symptom be used to diagnose an individual with Autism Spectrum Disorder? - Insistence on sameness • **Yes** • No	“the phrase "insistence on sameness" feels like jargon/not in plain language”
		“not on its own though - could be interpreted as meaning it is sufficient - should clarify contribute towards diagnosis”
		“It should not be used on it's own to diagnose, but the answer is relevant to ask when assessing a child. so, "as part of an assessment" would be better”
		“It presupposes that the respondent is fully conversant with the concept of 'sameness' as it is used in the assessment and diagnosis of autism as this is different from the everyday meaning of sameness with its associations with bland, dull, uninteresting, etc”
McMahon et al., 2020 (#15)	Can this symptom be used to diagnose an individual with Autism Spectrum Disorder? - Failure of normal back-and-forth conversation • **Yes** • No	“Need more than 1 symptom to diagnose someone”
		“It's just always hard to have "normal" for social communication expectations. This skill varies based on situation, culture, and even interest in the topic”
		“Here the ambiguity lies in the details how long of a back and forth? The term "back and forth conversation" should be defined.”
		““Failure of normal back-and-forth conversation” Is very vague. What counts as a failure? What counts as a normal conversation? And "back-and forth conversation" requires two conversationalists, by definition”

Note. “Correct” answers, as provided by the assessment authors, are bolded

#### Theme #1: Disagreement/Lack of Clarity in the Research Literature

 Several autism experts identified questions wherein the research literature was mixed, and the answer indicated to be “correct” for the autism knowledge question oversimplified or did not account for new or variable results in the research literature. Some autism experts cited specific studies that refuted the “correct” answer provided on an autism knowledge questionnaire. For instance: (1) a systematic review showing that equine therapy has beneficial effects for individuals with autism suggests that it can be considered an evidence-based intervention (Srinivasan et al., 2018; #10 on McClain et al., 2020); (2) a meta-analysis examining variables with low risk of bias in randomized controlled trials suggests that early interventions for autistic children may have limited efficacy (Sandbank et al., 2020; #22 on Harrison et al., 2017a^3^); and (3) another meta-analysis indicating that the male-to-female ratio in autism is closer to 3:1 due to under-diagnosis of females suggests that the often-cited 4:1 ratio may not be accurate (Loomes et al., 2017; #2 on McClain et al., 2020). Autism experts also identified several autism knowledge questions for which people might hold different perspectives, such as whether autism should be classified as a communication disorder or a neurodevelopmental disability with communication difficulties (#35 on Harrison et al., 2017a), whether behavioral therapy should be categorized as effective given that many autistic individuals do not report positive experiences with behavioral therapy (#38 on Harrison et al., 2017a), and whether children who received an autism diagnosis will necessarily maintain that diagnosis in adulthood (#10 on Gillespie-Lynch et al., 2015).

#### Theme #2: Not Fully Acknowledging the Heterogeneity of the Autism Spectrum

 Autism experts also identified several questions that were true of some autistic individuals, but not true of every individual with autism. For instance, while some autistic individuals will attend college and/or marry, many individuals with autism will not (#4 on Gillespie-Lynch et al., 2015); while some autistic individuals may need special education services at school, other individuals with autism will not (#9 on Harrison et al., 2017a); and while some children can be diagnosed with autism at two years of age, other children have more subtle autistic characteristics and may not fully meet the diagnostic criteria for autism until a later age (#22 on McClain et al., 2020). In addition, several autism experts expressed concern about questions seemingly aimed to curtail stereotypes or myths about autism, such as those indicating that individuals with autism show affection (#8 on Gillespie-Lynch et al., 2015), are not violent (#11 on Gillespie-Lynch et al., 2015), and are interested in making friends (#12 on Gillespie-Lynch et al., 2015). While these questions can combat autism-related stereotypes, these questions may also portray autistic individuals as a monolithic and homogeneous group. Both individuals with and without autism can have difficulty showing affection, can be violent, and can be disinterested in friendship. As such, autism experts were concerned that the heterogeneity of the autism spectrum was not fully represented by some autism knowledge questions.

#### Theme #3: Lack of Clarity in the Question/Answer Prompt

 Autism experts identified several ways that autism knowledge questions lacked clarity, potentially causing confusion about how to understand or respond to the question. For instance, several autism knowledge questions used imprecise terminology when referring to proportions of autistic individuals, such as “a lot of children with autism” (#41 on Harrison et al., 2017a) and “many children with autism” (#34 on Harrison et al., 2017a). Terms such as “everyday language” (#44 on Harrison et al., 2017a) and “proper treatment” (#7 on Gillespie-Lynch et al., 2015) are vague and could be interpreted differently across different members of the autism community. Also, terminology taken directly from the DSM-5 (American Psychiatric Association, 2013, 2022), such as “insistence on sameness” (#9 on McMahon et al., 2020), “inflexible adherence to routines” (#22 on McMahon et al., 2020), and “poorly integrated verbal and nonverbal communication” (#4 on McMahon et al., 2020), may have been too technical for a non-professional audience. In addition, although such terminology is taken from the DSM-5, phrases such as “abnormalities in eye contact and body language” (#19 on McMahon et al., 2020) and “failure of normal back-and-forth conversation” (#15 on McMahon et al., 2020) can be perceived as stigmatizing and ableist. Other questions presented ambiguity in content, such as asking whether a child with autism can follow a person’s point, without specifying the age of the child (#28 on Harrison et al., 2017a) and asking about autism prevalence rates internationally, without acknowledge that diagnosis rates may differ from true prevalence rates (#24 on McClain et al., 2020). A few questions raised concern about cultural and religious bias, including a question about special education in the United States, which may not be appropriate for international respondents (#8 on McClain et al., 2020), and a question about God causing autism, which may assess religious beliefs rather than scientific knowledge (#51 on Harrison et al., 2017a). Finally, the general format for two of the questionnaires led to some confusion across participants. For the Gillespie-Lynch et al. (2015) assessment, the 5-point Likert scale was perceived to be an unusual response format for a knowledge questionnaire. For the McMahon et al. (2020) assessment, greater clarity was needed to show that the autistic symptom in the question prompt was one of several symptoms that could be used to diagnose autism, not the singular symptom that should be used to diagnose autism.^4^

## Discussion

The current study examined the substantive validity of four autism knowledge assessments with existing psychometric support (Gillespie-Lynch et al., 2015; Harrison et al., 2017a; McClain et al., 2020; McMahon et al., 2020). Feedback from autism experts suggests that 34% of the questions on these assessments should be evaluated more closely for accuracy and 17% of the questions should be evaluated more closely for ambiguity. While a sizeable number of questions were flagged as potentially problematic for accuracy and/or ambiguity, it is also worth noting that the majority of autism knowledge questions (62% of the questions) did not raise concerns about accuracy or ambiguity. As such, while these autism knowledge assessments can and should be improved, they primarily consist of well-worded, clear questions that validly assess autism knowledge.

Overall, autism experts identified three main areas of improvement for autism knowledge questions. First, such questions should accurately represent the current research literature and be cautious about making definitive conclusions when the research literature is mixed or still evolving. For instance, although most children with autism do grow up to be adults with autism (#10 on Gillespie-Lynch et al., 2015), research shows that not all children with autism continue to meet the diagnostic criteria for autism in adulthood (Fein et al., 2013). There are several reasons why autistic children may not maintain their diagnosis in adulthood (Jellett & Muggleton, 2022), including camouflaging autistic characteristics to appear neurotypical and building a supportive environment wherein autistic characteristics do not lead to “clinically significant impairment” (American Psychiatric Association, 2022). As such, autism knowledge questions need to be examined carefully, as even questions that may seem intuitive or straightforward can contain important nuances that affect validity. Moreover, as autism research progresses, it is important to regularly re-evaluate the validity of autism knowledge questions. Questions that were considered previously valid may not retain their validity in future years.

Second, questions on autism knowledge measures should acknowledge the heterogeneity across the spectrum. Autism knowledge questions that are true for some individuals with autism, but not true for other autistic individuals, may be biased toward or against specific subgroups within the autism community (e.g., autistic individuals with or without an intellectual disability). Also, while autism knowledge questions can be used to counter autism stereotypes or misconceptions, such questions should still recognize heterogeneity and individual differences within the autism spectrum, so as not to portray individuals with autism as a monolithic, homogeneous group.

Third, autism knowledge questions should use clear and precise language to minimize ambiguity. Some terminology may be too technical for a lay audience; additionally, some terminology, including scientific or clinical terminology, may have stigmatizing or ableist connotations. The language used in autism knowledge questions should be scientifically accurate, yet also minimize stigma. See Bottema-Beutel et al. (2021) and Singer et al. (2022) for more discussion and debate about the appropriate language in describing autism. For some autism knowledge questions, more context or specificity may be needed to reduce ambiguity (e.g., providing an age or age range when discussing social development milestones). Also, unless an autism knowledge assessment is purposefully designed to index autism knowledge in a specific subgroup, questions should minimize bias across different groups of potential respondents (e.g., American and international respondents, respondents with and without autism, religious and atheist respondents).

### Recommendations for Using the Assessments in Future Research

Authors of the four autism knowledge assessments examined in this study are encouraged to review the list of potentially problematic questions for their assessment and consider removing or revising these questions. Additionally, even those questions that were not flagged as “potentially problematic” should be evaluated for improvement, as some expert reviewers identified concerns with these questions. For example, a question referencing a “cure” for autism should consider that not all autism influencers desire a “cure”, as well as the potential ambiguity between “cure”, improved skills, and a positive outcome (#12 on Harrison et al., 2017a). As another example, a question asking about frequency and timing of diagnosis across racial and ethnic groups should consider that current prevalence data does not seem to be consistent with historical diagnostic trends (Maenner et al., 2023; #4 on McClain et al., 2020).

Until revised versions of autism knowledge assessments are published, we provide the following practical recommendations for continued use of these assessments in the research literature:

1. If a question was not flagged for ambiguity or accuracy, that question has been evaluated by autism experts and the substantive validity of that question has been confirmed. These questions can be used with relative confidence in future research, although such questions should still be examined by assessment authors for potential improvement and need to be re-evaluated for accuracy as new research literature emerges.
2. If a question has been flagged as potentially problematic for both ambiguity and accuracy, we recommend deleting that question from the assessment. Similarly, if less than 80% of autism experts provided the “correct” response to the assessment, we recommend deleting the question from the assessment, regardless of its ambiguity rating. In the future, these questions may be revised by the assessment authors to address any problematic components, but until such revisions occur, we suggest using the assessments without these questions. The questions that fit the above criteria are #6, 8, 11, and 13 on the Gillespie-Lynch et al. (2015, 2019) assessment, #9, 20, 28, 35, 38, and 41 on the Harrison et al. (2017a) assessment, #5, 12, 17, 21, 22, 23, 24, and 25 on the McClain et al. (2020) assessment, and #4 on the McMahon et al. (2020) assessment.
3. If a question was flagged as potentially problematic for either accuracy or ambiguity, and does not meet the exclusion criteria described above, we recommend that researchers carefully examine and evaluate that question before including it in the assessment. Not every flagged question is necessarily problematic, and flagged questions may only be problematic in specific contexts. For instance, a question on special education in the United States (#8 on McClain et al., 2020) might be deleted if conducting research internationally, but might be retained if conducting research in the United States. The questions that were flagged as potentially problematic, but do not meet the exclusion criteria outlined previously, are #3, 4, 7, 9, 10, and 12 on the Gillespie-Lynch et al. (2015, 2019) assessment, #22, 34, 36, 44, and 51 on the Harrison et al. (2017a) assessment, #1, 2, 3, 8, 10, 13, and 20 on the McClain et al. (2020) assessment, and #3, 9, 15, 19, and 22 on the McMahon et al. (2020) assessment.
4. As several respondents expressed confusion with the 5-point Likert scale response format on the Gillespie-Lynch et al. (2015, 2019) assessment, we suggest using an agree/disagree or true/false response format for this assessment. Also, we recommend that the McMahon et al. (2020) assessment be administered in a checklist format, as originally intended, to clarify that participants are evaluating whether the symptom is one of several symptoms that can be used to diagnose autism. The assessment directions can also be modified as follows for clarity: “Is this one of the symptoms that can be used to diagnose an individual with Autism Spectrum Disorder?”
5. When these assessments are used in future research, we encourage researchers to clearly note any changes that were made to the original assessment (e.g., questions deleted, different response format). The psychometric properties of the modified assessment, such as internal consistency, should be analyzed and reported in any resulting manuscripts.

Finally, while the four autism knowledge assessments evaluated in this study can be improved, it is important to note that we are not discouraging use of these assessments in future research. These assessments have undergone a rigorous substantive validity review by autism experts, and this review has provided more clarity as to which assessment questions may need to be deleted, modified, or examined more closely in the future. This feedback from autism experts can and should be used to improve the assessments, and both short-term modifications made by researchers actively using these assessments and more permanent revisions from assessment authors will enhance the validity of these assessments.

### Broader Implications for Autism Knowledge Research

In addition to providing valuable feedback on the validity of the four autism knowledge assessments examined in this study (Gillespie-Lynch et al., 2015; Harrison et al., 2017a; McClain et al., 2020; McMahon et al., 2020), the expert feedback from this study provides critical insight into the broader study of autism knowledge. When conducting autism knowledge research, it is essential to differentiate between facts and theoretical perspectives. Within autism research, some information is factual, such as the current criteria for diagnosing autism in the DSM-5-TR (American Psychiatric Association, 2022). While the diagnostic criteria will likely change in the future, and different autism experts and influencers may disagree on how the criteria should change, there are currently established guidelines for diagnosing autism. Conversely, other autism-related information is opinion-based, such that different experts and influencers may hold different theoretical perspectives. For instance, while the Autistic Self Advocacy Network (2024) does not believe that sheltered workshops should be a vocational option for adults with autism, the National Council on Severe Autism (2024) advocates for their existence. Questions on autism knowledge measures should assess factual information. If there is reasonable disagreement as to an autism statement, it is an opinion- or theory-based statement, rather than a fact-based statement.

Relatedly, authors of autism knowledge assessments should be aware of their own biases, opinions, and theoretical perspectives when creating or revising autism knowledge questions. For an autism knowledge question to be fact-based, a diverse set of experts with access to different types of autism knowledge should agree that the question and answer are fact-based. Depending on the specific content knowledge being evaluated, an appropriate panel of experts could include individuals with diverse personal expertise in autism (e.g., autistic self-advocates, parents of individuals with severe autism) and diverse professional expertise in autism (e.g., clinical psychologists, special education teachers, neuroscientists). Finally, it is worth noting that acknowledging a question/answer as fact-based is not the same as personally endorsing or approving of that fact. For instance, an autism influencer might agree that “abnormalities in eye contact and body language” is currently included as a symptom of autism in the DSM-5-TR (American Psychiatric Association, 2022), and simultaneously, might strongly advocate for a change in the diagnostic criteria and language in the future DSM-6.

This feedback from autism experts also underscores the difficulty in writing accurate, fact-based autism knowledge questions, given the heterogeneity of the autism spectrum. Over the past several decades, there has been considerable debate about whether autism should be a unified spectrum or separated into different diagnoses. In the DSM-IV, the autism spectrum was subdivided into different diagnoses, most notably Asperger’s Disorder and autistic disorder (American Psychiatric Association, 1994, 2000). In the DSM-5 (American Psychiatric Association, 2013, 2022), the various autism-related diagnoses were unified into a single diagnosis of autism spectrum disorder. In the proposed DSM-6, there is debate about whether the autism spectrum should again be subdivided, with a new potential diagnosis of profound autism being discussed (Lord et al., 2022). When writing and revising autism knowledge questions, authors of autism knowledge assessments must carefully consider these different subgroups on the autism spectrum. If a question does not seem equally applicable and appropriate to individuals who might have formerly received an Asperger’s Disorder diagnosis in the DSM-IV (American Psychiatric Association, 1994, 2000) *and* to individuals who fit the proposed criteria for profound autism (Lord et al., 2022), the question may be biased toward or against a subgroup of autistic individuals. Alternatively, authors of autism knowledge assessments could also consider designing questionnaires to measure autism knowledge of specific, well-defined subgroups on the autism spectrum (e.g., autistic individuals attending college), such that knowledge questions wouldn’t need to be representative of the full autism spectrum.

### Limitations

In this study, the pool of expert reviewers was relatively small, with about 30 autism experts evaluating each question (see Table 2). In addition, participants were only eligible to be an expert reviewer if they served on the editorial board of a peer-reviewed autism journal. This inclusion criteria ensured that experts had the clinical and scientific expertise to evaluate the accuracy of autism knowledge questions. However, given this inclusion criteria, the feedback of individuals with professional and/or personal expertise in autism, but who did not also serve on the editorial board of an autism journal (e.g., autistic individuals, parents, teachers, clinicians), was not solicited for the current study. Additionally, cognitive interviewing with individuals in the general population, without expertise in autism, was not conducted in this study, and such interviewing likely would have revealed additional ambiguities in the question/answer prompts. Also, autism experts reported the most familiarity with Severity Level 1 (“requiring support”) and the least familiarity with Severity Level 3 (“requiring very substantial support”; American Psychiatric Association, 2022), such that experts may have been more likely to consider accuracy/ambiguity for individuals with autism requiring support (Level 1) in their ratings and qualitative comments, compared to individuals with autism requiring very substantial support (Level 3). Finally, as the autism knowledge assessments examined in this study vary in terms of content difficulty, intended audience, and response format, direct comparisons cannot be made across the accuracy/ambiguity ratings. A higher ambiguity rating or a lower accuracy rating does not necessarily index a poor question; rather, it may index a more complex question or a question targeted toward a professional audience who has received specialized autism training.

### Future Directions

In future research, the four autism knowledge assessments targeted in this study (Gillespie-Lynch et al., 2015; Harrison et al., 2017a; McClain et al., 2020; McMahon et al., 2020) should be revised in response to this expert feedback, with the psychometric properties of these assessments reevaluated. When developing new autism knowledge assessments, substantive validity should be carefully considered, with feedback solicited from a diverse group of autism experts and/or potential respondents. In particular, new autism knowledge assessments should be reviewed to determine whether they accurately represent the current research literature, acknowledge the heterogeneity of the autism spectrum, and use clear and precise language in the question/answer prompt.

## Footnotes

1 As research suggests that different individuals in the autism community have different preferences regarding identity-first and person-first language (e.g., Kenny et al., 2016), the current paper uses both identity-first and person-first language, an approach recommended by Dunn & Andrews (2015).

2 In their explanations, a few participants indicated that they wished to change prior responses. However, as it was difficult to determine whether these requests were due to a legitimate mistake in selecting an unintended response or whether additional information about the “correct” answer from the assessment authors influenced their interpretation of the question, all original responses were retained.

3 The numbers presented in this paper for the Autism Stigma and Knowledge Questionnaire are consistent with the numbers provided in Table 9 of Harrison et al. (2017a). Note that this numbering is not the final numbering for the questionnaire, though, as some initial questions were excluded from the final version of the questionnaire.

4 In the original McMahon et al. (2020) assessment, participants were given a checklist of symptoms and asked to indicate whether each symptom on the checklist can be used to diagnose an individual with autism. As symptoms were presented singularly in the current study, rather than together in a checklist format, this altered presentation style may have led to confusion about whether symptoms could be used singularly to diagnose autism.
